# Food insecurity and SNAP use among sexual minority people: analysis of a population-based sample from National Health Interview Survey, 2017

**DOI:** 10.1186/s12889-022-13391-7

**Published:** 2022-05-13

**Authors:** Jennifer M. Jabson Tree, Jennifer Russomanno, Marissa Bartmess, Joel G. Anderson

**Affiliations:** 1 Department of Public Health, University of Tennessee, 1914 Andy Holt Ave, 390 HPER, Knoxville, TN 37996 USA; 2 College of Nursing, University of Tennessee, 1200 Volunteer Blvd, Knoxville, TN 37916 USA

**Keywords:** Food insecurity, Public health, Sexual minorities, Food assistance, Homosexuality

## Abstract

**Background:**

Food insecurity is a pressing public health problem. Lesbian, gay, and bisexual (LGB) people are at increased risk for food insecurity, yet this issue remains grossly understudied among this population. The purpose of this study was to add to the existing literature surrounding food insecurity and the use of federal food assistance programs (SNAP) among LGB people.

**Methods:**

This study used publicly available, de-identified data from the 2017 National Health Interview Survey (NHIS). Primary variables were sexual orientation, food security status, and receipt of SNAP. Food security was assessed using the 10-item USDA Family Food Security measure.

**Results:**

In our sample, people who identified as bisexual had the highest rates of food insecurity (23.8%, *n* = 76). Female sexual minorities were 52% more likely to experience food insecurity (aOR = 1.518, 95% CI 1.105–2.087, *p* = .01) and 44% more likely to report household SNAP assistance than their heterosexual counterparts (aOR = 1.441, 95% CI 1.025–2.028, *p* = .03). SNAP partially mediated the association between sexual orientation and food insecurity for LGB females.

**Conclusions:**

Our findings add to the growing empirical evidence documenting food insecurities among sexual minority adults. Our results reiterate the need for sexual orientation to be included in nationally representative federal food security measures.

## Background

All humans have the basic human right to adequate food [1]. Yet not all humans have equitable access to food and often experience food insecurity. Food insecurity is defined by the United States Department of Agriculture (USDA) as a “lack of access to enough food necessary for each member of a household to lead an active, healthy lifestyle” [2]. In 2019, approximately 10.5% of U.S. households (35.2 million people) were classified as food insecure [2]. Food insecurity has been linked to a number of adverse health conditions including anemia, asthma, diabetes, cancer, chronic obstructive pulmonary disease (COPD), and heart disease, [3] as well as mental health issues including depression and stress [4].

The risk for and experience of food insecurity is not equally experienced by all demographic groups, and some are more likely to be food insecure than others. For example, people with incomes below 185% of the Federal Poverty Level (FPL) are at greater risk for food insecurity, and low-income households headed by single women have higher rates of food insecurity (40.8%) than two-parent households (34.8%) [5].

Groups that face social bias and structural oppression, such as—but not limited to—heterosexism, are at risk of experiencing food insecurity. For the purposes of this work, heterosexism is defined as a social system of inequity where structural- and individual-level discrimination or prejudice exists against gay people and assumes that heterosexuality is the only normal form of sexual orientation [6]. Lesbian, gay, and bisexual (LGB) people are a group that face minority stressors in the form of social bias and structural oppression and discrimination that may contribute to their risk for food insecurity [7, 8]. Fredriksen-Goldsen et al., [9] provide a life course framework that describes multi-level mechanisms for how LGB people may come to experience greater food insecurity than heterosexual people. The Health Equity Promotion Model outlines multi-level contextual factors and health-promoting and adverse pathways that all relate to health outcomes such as food insecurity. This multi-level context is comprised of structural- and individual-level pathways that influence food insecurity disparities. Structural-level pathways are social exclusion, social stigma, and institutional heterosexism. These factors influence LGB people’s ability to secure and maintain employment, earn equitable and livable wages, and access resources that may relieve food insecurity. For example, if a state does not include LGB identities in state-level anti-discrimination laws, then employers can discriminate against LGB employees, and this can increase the likelihood of food insecurity for LGB people. At the individual employer level, if an employer has policies that prevent hiring an individual with a known LGB identity, or policies that allow termination of employees with LGB identities, these also can promote food insecurity for LGB people. Individual-level pathways include microaggressions, discrimination, and abuse that occurs between individuals. These pathways may also diminish LGB people’s ability to maintain and secure employment. For example, if a supervisor holds anti-LGB ideologies and learns that an employee holds an LGB identity, the employee may be microaggressed, terminated, overlooked for advancement opportunities, or may leave an employment position because of microaggressions, all of which could result in food insecurity for LGB people.

According to Fredriksen-Goldsen et al. [9], the multi-level context of the Health Equity Promotion Model relates to health-promoting and adverse pathways, which include social and community structures. These also contribute to food insecurity for LGB people through social isolation and lack of family supports during financially unstable periods. LGB individuals very often are rejected by family of origin in the coming out process. Therefore, older or more financially secure family members who might otherwise be expected to assist with alleviating food insecurity for family members are unavailable due to an individual’s LGB identity.

Yet, despite strong theoretical support for the idea that LGB people may be more likely to experience food insecurity, to our knowledge, there are currently very few empirical, peer-reviewed publications that report on the experiences of LGB people regarding food insecurity. We identified three such publications. Testa and colleagues [10] and Gibb and colleagues [11] reported that relative to heterosexual individuals, bisexual individuals had significantly higher rates of mild and moderate-to-severe food insecurity compared to heterosexual individuals. Similarly, in their population-based study using data from the National Health and Nutrition Examination Survey (NHANES), Patterson and colleagues [8] found that lesbian women were 52% more likely to experience food insecurity than their heterosexual counterparts (aOR = 1.52, 95% CI 1.05–2.20, *p* < .001), and bisexual women were 34% more likely to experience food insecurity than the referent heterosexual group (aOR = 1.34, 95% CI 1.05–1.70, *p* < .001). Gibb and colleagues [11] also reported that lesbian/gay individuals experienced higher rates of severe food insecurity (13.14, 95% CI 10.07, 16.97) than heterosexual individuals.

To quell the number of food insecure households, the U.S. implements several safeguards against food insecurity, the largest of which is the Supplemental Nutrition Assistance Program (SNAP), formerly known as food stamps. In 2018, SNAP served approximately 40 million households with an annual expenditure of $68 billion. SNAP provides monthly, income-based monetary benefits to assist households with securing needed food supplies. To be SNAP-eligible, households must meet several requirements related to household resources, economic means, and incomes. In general, a household may qualify for SNAP if its gross monthly income does not exceed 130% of the FPL, commiserate with the household’s size [12].

There is very limited peer-reviewed, empirical evidence concerning SNAP usage by LGB people. The only peer-reviewed publication, to our knowledge, that reported on SNAP utilization used data from NHANES to investigate differences in SNAP usage among lesbian and bisexual women and did not report on SNAP usage among gay and bisexual men [13]. In their publication, Patterson and colleagues [8] did not find any differences in SNAP usage by lesbian and bisexual women as compared to heterosexual women. Additionally, although SNAP was designed and implemented to reduce food insecurity, it is not yet empirically documented by the peer-reviewed literature if this is true for the LGB population. Evidence from research with the general population suggests there may be a nuanced relationship between SNAP and the alleviation of food insecurity for all populations [14]. Specifically, SNAP does not fully alleviate food insecurity for all sub-populations but the full conditions of this have not yet been fully investigated. How SNAP relates to food insecurity for LGB people has not yet been investigated or documented.

Access to adequate food is a basic human right that should be made available to everyone [1, 15]. Food insecurity among LGB people is preventable. However, without documenting where we are in relation to food insecurity among LGB people, we are unable to identify and deploy appropriate public health interventions. Therefore, the purpose of the current study is to add to the existing literature concerning food insecurity and SNAP usage among LGB people using data from the 2017 National Health Interview Survey (NHIS). NHIS is one of the few population-based surveys that measures sexual orientation, SNAP usage, and food insecurity. Based on previous evidence, we hypothesize that food insecurity and SNAP usage varies by sexual orientation, with LGB people reporting greater odds for food insecurity and receiving SNAP than heterosexual people. We also hypothesize that SNAP usage will reduce food insecurity.

## Methods

This study involved secondary analyses of publicly available, de-identified data and did not require human subjects review. The analytic protocol was performed in accordance with relevant guidelines and regulations.

### Survey description

Data for this study were from the 2017 NHIS. The NHIS is a national, representative, probability, cross-sectional interview survey of U.S. households. The survey assesses basic health and demographic information for all household members. Detailed information concerning the NHIS sampling frame and study design is described elsewhere [16].

### Sample

Casewise deletion was used for missing data related to variables of interest (described below). A total of 3283 observations were omitted, the majority of which were respondents for whom sexual orientation (*n* = 1107) or income (*n* = 1990) data were missing or refused to answer. Excluded cases (i.e., those with missing values for the variables of interest) were compared with included cases to determine any differences in terms of sex, age, and ethnicity given that these were the only demographic variables without missing values. Excluded cases were significantly younger (included cases = 50.4 ± 18.5 years, excluded cases = 54.9 ± 18.9 years; *p* < 0.001) and included significantly more female respondents (included cases = 54.4%, excluded cases = 57.3%; *p* = 0.002). Statistical analyses were adjusted for these two variables (described below). There was no difference in terms of ethnicity (*p* = 0.911). The final unweighted sample size was *N* = 23,459: *n* = 201 (lesbian), *n* = 253 (gay), *n* = 320 (bisexual), and *n* = 22,685 (heterosexual).

### Measures

#### Food security

Food security was assessed with the 10-item USDA Family Food Security measure [2] addressing adult 30-day food security. The content of the questions includes, but is not limited to, items that evaluate being worried food will not last, eating less than one should, being hungry but not eating, and cutting or skipping meals within the previous 30 days. Responses range from 0 to 10 with higher scores indicating lower food security. A dichotomous food security variable (0 = food secure, 1 = food insecure) was created from raw food security scores from the ten questions on the Family Food Security Supplement (FFS section) of the NHIS. Continuous food security raw scores were categorized as food secure (raw scores of 0–2), low food security (raw scores of 3–5), and very low food security (raw scores of 6–10), which were then combined to create the final dichotomous variable of *food security* (raw scores of 0–2 coded as “0= food secure” and raw scores of 3–10 coded as “1= food insecure”). The decision to dichotomize the variable was based on the NHIS cutoff scores published by the National Center for Health Statistics (2018).

#### Receipt of SNAP

Receipt of SNAP was assessed with one question and responses were binary: “Have you or anyone in your family received SNAP assistance in the past 12 months?” Respondents affirming that they or another household member received food stamps/SNAP benefits in the previous 12 months were coded as receiving SNAP (1) versus those who did not receive previous 12-month SNAP benefits (0).

#### Sexual orientation

Sexual orientation was assessed with a single sexual orientation identity question [17] for men and women: “Which of the following best represents how you think of yourself?” Response options included gay, straight, bisexual, something else, or I do not know. Women who self-identified as gay were labeled ‘lesbian’. Respondents who selected ‘something else’ or ‘I do not know’ were excluded from analyses. Heterosexual sexual orientation was the referent category.

#### Demographic characteristics

Demographic characteristics used to describe the sample included sex (female or male), age, race/ethnicity, marital status, employment status, income, chronic disease (having at least one or none), current smoking status (smoker or non-smoker), and general health status where respondents were asked to compare their health as “better” (0), “worse” (1) or “about the same” (2) as 12 months ago. Demographic covariates used to adjust multiple-variable models were selected based on theoretical and empirical evidence. Covariates included race, marital status, age, employment status, chronic disease (having at least one or none), current smoking status (smoker or non-smoker), and general health status. Description of assessment of demographic characteristics is provided by NHIS [16].

### Statistical analyses

Categorical variables are presented in terms of frequencies and percentages. Chi-squared tests were conducted to examine associations between the categorical variables. Using NHIS survey sample weights, binary logistic regression models were used to determine if there were predictive relationships between sociodemographic variables, food security, and SNAP status. To assess differences in food security and SNAP status among the sample better and to address differences between included and excluded cases, the regression models were stratified by sex (*n* = 10,707 for male-only regression models and *n* = 12,752 for female-only regression models) and age (*n* = 11,174 for younger-adult-only [aged 18–49] regression models and *n* = 12,285 for older-adult-only [aged ≥50 years] regression models). To test how SNAP related to food insecurity, mediation analyses were conducted. Mediation effects were assessed using the Sobel test [18]. This included testing associations between sexual orientation and food insecurity, sexual orientation and SNAP utilization, and the change in association between sexual orientation and food insecurity in the presence of the SNAP variable [19]. Weighted regression analysis results are presented in terms of odds ratios. All analyses were completed using IBM SPSS statistics (Version 26).

## Results

### Participants

Study sample demographic characteristics are outlined in Table 1. Most NHIS respondents (96.7%, *n* = 22,685) identified as heterosexual, with 3.3% (*n* = 774) identifying as LGB. Of those who identified as LGB, 26% (*n* = 201) identified as lesbian, 32.7% (*n* = 253) identified as gay, and 41.3% (*n* = 320) identified as bisexual. Overall, NHIS respondents were represented across all age ranges; however, most respondents who identified as bisexual were aged 18–34 (59%, *n* = 189). A majority of respondents, regardless of sexual orientation, identified as white (80.6%, *n* = 18,909), with smaller percentages identifying as a racial minority, including Asian (5.2%, *n* = 1209), American Indian or Native American (AINAN) (1.1%, *n* = 264), Black (10.9%, *n* = 2550), or multiple races (2.1%, *n* = 483).

**Table 1:** Sample characteristics in study sample by self-reported sexual orientation: NHIS 2017 (*n* = 23,459)

	Gay (*n* = 253)	Lesbian (*n* = 201)	Bisexual (*n* = 320)	Heterosexual (*n* = 22,685)	x^2^	*p*
	% (n)^a^					
Sex					517.08	<.001
Male	100.0 (253)	0.0 (0)	26.6 (85)	45.7 (10,369)		
Female	0.0 (0)	100.0 (201)	73.4 (235)	54.3 (12,316)		
Age					264.80	<.001
18–34	34.8 (88)	25.9 (52)	59.0 (189)	24.3 (5529)		
35–49	21.3 (54)	29.8 (60)	18.2 (58)	22.7 (5144)		
50–64	33.2 (84)	26.9 (54)	15.6 (50)	26.1 (5913)		
65+	10.7 (27)	17.4 (35)	7.2 (23)	26.9 (6099)		
Race					59.08	<.001
Asian	4.0 (8)	2.0 (5)	2.8 (9)	5.2 (1187)		
Black	7.1 (18)	10.9 (22)	10.6 (34)	10.9 (2476)		
AINAN	2.0 (4)	0.8 (2)	0.9 (3)	1.1 (255)		
White	85.8 (217)	80.1 (161)	78.8 (252)	80.6 (18,279)		
Multiple Race	4.0 (10)	3.0 (6)	6.9 (22)	2.0 (445)		
Ethnicity					0.93	0.818
Non-Latinx	86.6 (219)	89.1 (179)	87.5 (280)	88.2 (20,007)		
Latinx	13.4 (34)	10.9 (22)	12.5 (40)	11.8 (2678)		
Geographical Region					17.35	0.044
Midwest	20.2 (51)	19.9 (40)	22.8 (73)	24.1 (5470)		
Northeast	16.2 (41)	14.9 (30)	20.0 (64)	15.8 (3595)		
South	35.6 (90)	34.8 (70)	31.3 (100)	36.8 (8346)		
West	28.1 (71)	30.3 (61)	25.9 (83)	23.2 (5274)		
Marital status					348.37	<.001
Partnered/Married	35.2 (89)	43.3 (87)	30.6 (98)	50.9 (11,541)		
Never married	54.2 (137)	41.3 (83)	49.7 (159)	21.8 (4953)		
Divorced/Separated	8.7 (22)	11.9 (24)	17.8 (57)	17.6 (3999)		
Widowed	2.0 (5)	3.5 (7)	1.9 (6)	9.7 (2192)		
Employment					31.97	<.001
Employed	72.7 (184)	64.2 (129)	67.8 (320)	58.9 (13,359)		
Unemployed	27.3 (69)	35.8 (72)	32.2 (103)	41.1 (9326)		
Income					67.59	<.001
$0–$34,999	29.6 (75)	40.8 (82)	54.1 (173)	34.3 (7781)		
$35,000–$74,999	31.2 (79)	23.9 (48)	24.1 (77)	30.1 (6822)		
$75,000–$99,000	15.0 (38)	13.9 (28)	6.3 (20)	11.5 (2611)		
$100,000+	24.1 (61)	21.4 (43)	15.6 (50)	24.1 (5471)		
Food security					53.15	<.001
Food secure	87.7 (222)	81.1 (163)	76.3 (244)	88.3 (20,039)		
Food insecure	12.3 (31)	18.9 (38)	23.8 (76)	11.7 (2646)		
Received SNAP					43.64	<.001
No	90.9 (230)	82.6 (166)	77.2 (247)	88.2 (20,001)		
Yes	9.1 (23)	17.4 (35)	22.8 (73)	11.8 (2684)		
Chronic disease					29.04	<.001
None	64.7 (130)	59.7 (151)	69.4 (222)	56.2 (12,744)		
At least one	40.3 (102)	35.3 (71)	30.6 (98)	43.8 (9941)		
Current smoker					30.43	<.001
Non-smoker	77.9 (197)	77.1 (155)	77.8 (249)	84.9 (19,261)		
Smoker	22.1 (56)	22.9 (46)	22.2 (71)	15.1 (3424)		
General health status					38.95	<.001
Better	20.6 (52)	17.9 (36)	30.6 (98)	19.1 (4327)		
Worse	7.1 (18)	10.4 (21)	12.5 (40)	8.8 (1990)		
About the same	72.3 (183)	71.6 (144)	56.9 (182)	72.2 (16,368)		

x^2^ = Pearson Chi-Square; *p* = *p*-value

^a^unweighted survey counts

### Food security

Unadjusted, bivariate analyses showed that respondents who identified as LGB had higher rates of food insecurity than those who identified as heterosexual (11.7%, *n* = 2646; Table 1). Among LGB people, those who identified as bisexual had the highest rates of food insecurity (23.8%, *n* = 76), followed by people who identified as gay (18.9%, *n* = 38) and lesbian (12.3%, *n* = 31).

Multivariable models were stratified by sex and adjusted for race, age, marital status, employment status, chronic disease, smoking status, and general health status (Table 2). In adjusted models, lesbian and bisexual women were 52% more likely to experience food insecurity than heterosexual women (aOR = 1.518, 95% CI = 1.105–2.087, *p* = .01). Food insecurity did not vary by sexual orientation among males.

**Table 2:** Demographic characteristics associated with food insecurity and SNAP receipt stratified by sex

	Food Insecurity								SNAP Receipt							
	95% Confidence Interval				95% Confidence Interval				95% Confidence Interval				95% Confidence Interval			
	aOR	LL	UP	*p*	aOR	LL	UP	*p*	aOR	LL	UP	*p*	aOR	LL	UP	*p*
	Males				Females				Males				Females			
Sexual Orientation																
Heterosexual	*ref*				*ref*				*ref*				*ref*			
LGB	1.211	0.789	1.859	0.381	1.518	1.105	2.087	**0.01**	1.119	0.697	1.794	0.642	1.441	1.025	2.028	**0.036**
Age	0.600	0.540	0.665	**<.001**	0.661	0.607	0.719	**<.001**	0.636	0.573	0.707	**<.001**	0.591	0.544	0.643	**<.001**
Race																
White	*ref*				*ref*				*ref*				*ref*			
Asian	0.479	0.290	0.792	**0.004**	1.333	0.942	1.886	0.105	0.919	0.605	1.397	0.694	0.909	0.634	1.303	0.604
Black	2.402	1.867	3.091	**<.001**	2.235	1.863	2.681	**<.001**	2.766	2.144	3.568	**<.001**	2.909	2.426	3.489	**<.001**
AINAN	1.990	1.060	3.737	**0.032**	2.100	1.298	3.395	**0.002**	3.793	1.944	7.401	**<.001**	2.814	1.701	4.653	**<.001**
Multiple Race	2.012	1.339	3.024	**0.001**	1.593	1.093	2.321	**0.015**	1.800	1.030	3.144	**0.039**	1.517	1.025	2.247	**0.037**
Marital status																
Partnered/Married	*ref*				*ref*				*ref*				*ref*			
Never married	1.058	1.840	1.333	0.633	1.538	1.272	1.860	**<.001**	0.790	0.619	1.010	0.060	1.686	1.386	2.052	**<.001**
Divorced/Separated	1.959	1.584	2.423	**<.001**	3.269	2.742	3.897	**<.001**	1.190	0.947	1.496	0.136	4.184	3.483	5.024	**<.001**
Widowed	1.259	0.840	1.888	0.264	1.555	1.217	1.987	**<.001**	1.089	0.947	1.703	0.707	2.343	1.782	3.081	**<.001**
Employment																
Employed	*ref*				*ref*				*ref*				*ref*			
Unemployed	2.396	1.955	2.938	**<.001**	2.038	1.749	2.374	**<.001**	3.148	2.554	3.881	**<.001**	1.834	2.415	3.326	**<.001**
Chronic disease																
None	*ref*				*ref*				*ref*				*ref*			
At least one	1.909	1.566	2.327	**<.001**	1.849	1.571	2.176	**<.001**	1.573	1.289	1.919	**<.001**	1.849	1.568	2.181	**<.001**
Current smoker																
Smoker	*ref*				*ref*				*ref*				*ref*			
Non-Smoker	0.414	0.344	0.500	**<.001**	0.380	0.323	0.447	**<.001**	0.341	0.282	0.413	**<.001**	0.356	0.304	0.418	**<.001**
General health status	0.955	0.861	1.059	0.383	0.944	0.868	1.026	0.176	1.030	0.925	1.147	0.591	0.941	0.863	1.025	0.161

Multivariable models were stratified by age and adjusted for sex, race, marital status, employment status, chronic disease, smoking status, and general health status (Table 3). In adjusted models, LGB young adults (i.e., 18–49 years) were 50% more likely to experience food insecurity than heterosexual young adults (aOR = 1.500, 95% CI = 1.184–1.899, *p* = .001). Food insecurity did not vary by sexual orientation among older adults. Females across the lifespan were more likely to experience food insecurity compared with males.

**Table 3:** Demographic characteristics associated with food insecurity and SNAP receipt stratified by age

	Food Insecurity								SNAP Receipt							
	95% Confidence Interval				95% Confidence Interval				95% Confidence Interval				95% Confidence Interval			
	aOR	LL	UP	*p*	aOR	LL	UP	*p*	aOR	LL	UP	*p*	aOR	LL	UP	*p*
	18–49 years				≥50 years				18–49 years				≥50 years			
Sexual Orientation																
Heterosexual	*ref*				*ref*				*ref*				*ref*			
LGB	1.500	1.184	1.899	**.001**	1.355	.932	1.969	.111	1.282	.995	1.651	.055	1.131	.764	1.675	.538
Sex																
Male	*ref*				*ref*				*ref*				*ref*			
Female	1.442	1.282	1.623	**<.001**	1.387	1.221	1.576	**<.001**	1.889	1.669	2.138	**<.001**	1.372	1.205	1.561	**<.001**
Race																
White	*ref*				*ref*				*ref*				*ref*			
Asian	.583	.427	.794	**.001**	1.595	1.160	2.191	**.004**	.460	.324	.651	**<.001**	2.263	1.684	3.042	**<.001**
Black	2.050	1.761	2.387	**<.001**	2.425	2.068	2.844	**<.001**	2.794	2.400	3.251	**<.001**	3.066	2.623	3.585	**<.001**
AINAN	1.691	1.126	2.538	**.011**	4.652	2.975	7.274	**<.001**	2.450	1.661	3.616	**<.001**	3.408	2.122	5.473	**<.001**
Multiple Race	1.676	1.229	2.286	**.001**	2.411	1.691	3.436	**<.001**	1.428	1.021	1.998	**.038**	2.443	1.701	3.509	**<.001**
Marital status																
Partnered/Married	*ref*				*ref*				*ref*				*ref*			
Never married	1.466	1.287	1.668	**<.001**	2.586	2.116	3.160	**<.001**	1.189	1.042	1.358	**.010**	4.120	3.376	5.027	**<.001**
Divorced/Separated	2.377	2.013	2.808	**<.001**	2.919	2.513	3.389	**<.001**	1.972	1.659	2.344	**<.001**	3.627	3.098	4.246	**<.001**
Widowed	1.811	1.008	3.253	**.047**	1.244	1.033	1.499	**.021**	1.266	.678	2.363	.459	1.718	1.427	2.068	**<.001**
Employment																
Employed	*ref*				*ref*				*ref*				*ref*			
Unemployed	2.044	1.805	2.314	**<.001**	2.118	1.841	2.435	**<.001**	3.254	2.878	3.679	**<.001**	3.019	2.599	3.507	**<.001**
Chronic disease																
None	*ref*				*ref*				*ref*				*ref*			
At least one	1.577	1.387	1.794	**<.001**	1.838	1.587	2.129	**<.001**	1.719	1.508	1.960	**<.001**	1.528	1.321	1.767	**<.001**
Current smoker																
Smoker	*ref*				*ref*				*ref*				*ref*			
Non-Smoker	.400	.351	.457	**<.001**	.349	.304	.402	**<.001**	.347	.303	.397	**<.001**	.355	.308	.410	**<.001**
General health status	.944	.883	1.010	.096	.857	.793	.925	**<.001**	.964	.899	1.033	.296	.870	.805	.942	**.001**

### Receipt of SNAP

When asked, “have you or anyone in your family received SNAP assistance in the past 12 months,” respondents who identified as gay (9.1%, *n* = 23; Table 1) reported the lowest rates of SNAP assistance. The highest receipt of SNAP was seen among respondents who identified as bisexual (22.8%, *n* = 73), followed by respondents who identified as lesbian (17.4%, *n* = 35) and those who identified as heterosexual (11.8%, *n* = 2684).

Multivariable models were stratified by sex and adjusted for race, age, marital status, employment status, chronic disease, smoking status, and general health status (Table 2). Lesbian and bisexual women were 44% more likely to report household SNAP assistance than heterosexual women (aOR = 1.441, 95% CI = 1.025–2.028, *p* = .03). Receipt of SNAP did not vary by sexual orientation for males.

Multivariable models were stratified by age and adjusted for sex, race, marital status, employment status, chronic disease, smoking status, and general health status (Table 3). Receipt of SNAP did not vary by sexual orientation when stratified by age. Females across the lifespan were more likely to report receipt of SNAP compared with males.

### Reduction of food insecurity by receipt of SNAP

For lesbian and bisexual females, we found evidence of partial mediation of the association between sexual orientation and food insecurity when SNAP receipt was entered into the model (Table 4). Among lesbian and bisexual females, the aOR for food insecurity (aOR 1.518, 95% CI = 1.105–2.087, *p* = .01) was reduced 9% with receipt of SNAP (aOR 1.388 95% CI = .98–1.95, *p* = .06). For females, there was a significant mediation effect of SNAP on the relationship between sexual orientation and food security (Sobel = 4.57; SE = 0.262; *p* < 0.001). For gay and bisexual males, there was no association between sexual orientation and food insecurity (aOR 1.21, 95% CI = .78–1.86; *p* = .38), nor sexual orientation and receipt of SNAP (aOR 1.119, 95% CI = .697–1.794; *p* = .64); therefore, the mediation analysis was terminated.

**Table 4:** Demographic characteristics associated with food insecurity after controlling for SNAP receipt stratified by sex

	Food Insecurity							
	95% Confidence Interval				95% Confidence Interval			
	aOR	LL	UP	*p*	aOR	LL	UP	*p*
	Males				Females			
Sexual Orientation								
Heterosexual	*ref*				*ref*			
LGB	1.173	0.776	1.773	0.448	1.388	0.989	1.949	0.058
Age	0.972	0.966	0.979	**<.001**	0.978	0.972	0.984	**<.001**
Race								
White	*ref*				*ref*			
Asian	0.475	0.291	0.778	**0.003**	1.403	0.980	2.007	0.064
Black	1.969	1.538	2.522	**<.001**	1.769	1.453	2.155	**<.001**
AINAN	1.488	0.779	2.843	0.229	1.620	0.915	2.867	0.098
Multiple Race	1.82	1.153	2.872	**0.01**	1.457	1.010	2.100	**0.044**
Marital status								
Partnered/Married	*ref*				*ref*			
Never married	1.053	0.826	1.342	0.676	1.309	1.072	1.599	**0.008**
Divorced/Separated	1.963	1.576	2.445	**<.001**	2.483	2.062	2.989	**<.001**
Widowed	1.348	0.849	2.141	0.205	1.473	1.138	1.906	**0.003**
Employment								
Employed	*ref*				*ref*			
Unemployed	1.869	1.526	2.289	**<.001**	1.639	1.395	1.925	**<.001**
Chronic disease								
None	*ref*				*ref*			
At least one	1.798	1.464	2.208	**<.001**	1.714	1.443	2.035	**<.001**
Current smoker								
Smoker	*ref*				*ref*			
Non-Smoker	0.514	0.423	0.624	**<.001**	0.477	0.398	0.57	**<.001**
General health status	0.946	0.851	1.051	0.299	0.958	0.876	1.047	0.346
SNAP receipt								
No	*ref*				*ref*			
Yes	0.213	0.17	0.268	**<.001**	0.245	0.208	0.288	**<.001**

For LGB people aged 18–49 years, there was evidence of partial mediation of the association between sexual orientation and food insecurity when SNAP was entered into the model (Table 5). Among LGB people aged 18–49 years, the aOR for food insecurity (aOR 1.50, 95% CI = 1.18–1.89; *p* = .001) was reduced 4% with receipt of SNAP (aOR 1.44, 95% CI = 1.13–1.85; *p* = .003). There was no association between sexual orientation and food insecurity for ages ≥50, nor sexual orientation and receipt of SNAP.

**Table 5:** Demographic characteristics associated with food insecurity after controlling for SNAP receipt stratified by age

	Food Insecurity							
	95% Confidence Interval				95% Confidence Interval			
	aOR	LL	UP	*p*	aOR	LL	UP	*p*
	18–49 years				≥50 years			
Sexual Orientation								
Heterosexual	*ref*				*ref*			
LGB	1.449	1.134	1.851	**.003**	1.352	.914	1.998	.131
Sex								
Male	*ref*				*ref*			
Female	1.260	1.116	1.423	**<.001**	1.281	1.122	1.463	**<.001**
Race								
White	*ref*				*ref*			
Asian	.656	.480	.897	**.008**	1.343	.966	1.869	.080
Black	1.671	1.426	1.959	**<.001**	1.859	1.568	2.205	**<.001**
AINAN	1.396	.914	2.133	.123	3.770	2.351	6.045	**<.001**
Multiple Race	1.594	1.156	2.198	**.004**	2.004	1.371	2.930	**<.001**
Marital status								
Partnered/Married	*ref*				*ref*			
Never married	1.431	1.254	1.634	**<.001**	1.896	1.535	2.342	**<.001**
Divorced/Separated	2.119	1.784	2.517	**<.001**	2.262	1.934	2.646	**<.001**
Widowed	1.785	.976	3.262	.060	1.138	.939	1.381	.188
Employment								
Employed	*ref*				*ref*			
Unemployed	1.565	1.371	1.785	**<.001**	1.659	1.435	1.919	**<.001**
Chronic disease								
None	*ref*				*ref*			
At least one	1.418	1.241	1.620	**<.001**	1.727	1.484	2.010	**<.001**
Current smoker								
Smoker	*ref*				*ref*			
Non-Smoker	.487	.425	.559	**<.001**	.433	.373	.503	**<.001**
General health status	.949	.885	1.017	.139	.880	.812	.955	**.002**
SNAP receipt								
No	*ref*				*ref*			
Yes	.258	.225	.296	**<.001**	.187	.162	.217	**<.001**

## Discussion

The Health Equity Promotion Model is multi-level framework that can help explicate how LGB people may come to experience inequity in food security across the life course. The multi-level context is comprised of structural- and individual-level factors [9] that confer stress and inequities through structural discrimination and interpersonal discrimination. These stressors influence LGB people’s employment, education, and other factors that contribute to economic stability (2–4; 9–12) and, subsequently, food security. Therefore, we anticipated that food insecurity and SNAP usage would vary by sexual orientation, where LGB people would report greater odds for food insecurity and receiving SNAP than heterosexual people. We also aimed to investigate the relationship between SNAP and food insecurity among LGB individuals. The purpose of this project was to add to the field by describing food insecurity, receipt of SNAP, and the influence SNAP had on food insecurity among LGB adults responding to a population-based health surveillance program.

Our analyses revealed evidence of disparities in food insecurity and SNAP use by sexual orientation. Lesbian and bisexual females had 52% greater odds of food insecurity and 44% greater odds of receiving SNAP than heterosexual females. While few studies exploring food insecurity and SNAP use among lesbian and bisexual females exist, our findings are consonant with the majority of the published evidence. Patterson and colleagues [8] demonstrated with their analysis of NHANES data that lesbian and bisexual females had 34–52% greater odds of experiencing food insecurity than heterosexual females. Additionally, in 2020, Testa and Jackson [20] reported that bisexual females were more likely to experience food insecurity than their heterosexual counterparts. In their study of adults participating in the 2017–2018 New York City community health survey, 40.7% of bisexual females experienced mild food insecurity and were twice as likely to experience mild food insecurity when compared to heterosexual females (RRR = 2.152, 95% CI = 3.13–3.527). It may be that lesbian and bisexual women’s marginalized identities at the intersection of sexism and heterosexism uniquely position them to be especially vulnerable to food insecurity. The most current census evidence indicates women earn 30% less than men [21]. The William’s Institute [22] reported that in the U.S., 25% of people with LGB identities report incomes equal to or less than $25,000 annually; this is 28% more than heterosexual individuals. Taken together, women who also identify as bisexual or lesbian are particularly vulnerable to food insecurity.

The models stratified by age indicated that LGB people 18–49 experienced more food insecurity compared to similarly aged heterosexual people. LGB people aged 18–49 also had higher rates of food insecurity than older LGB people. This may be explained by negative social consequences, such as rejection and isolation, that many LGB people experience when coming out. LGB people are coming out more frequently at younger ages [23], yet upon coming out, many LGB people experience rejection from family [24] that may contribute to economic instability, houselessness, and food insecurity.

There was no evidence of food insecurity among gay or bisexual males in this sample. This aligns with the limited available research concerning male- and female-led heads of households. In 2016, the USDA reported that single-parent, female-headed households were significantly more likely to be food insecure than single-parent, male-headed households (31.6% versus 21.7%) [5]. This could be, in part, due to wage gaps experienced by female workers, who, on average, earn 82 cents for every dollar paid to men [25]. Additionally, Matheson and McIntyre [26] uncovered that increased food insecurity was reported by single-female-led households as well as married/cohabitating households in which the survey respondent was female compared to male-led single households or male survey respondents of married/cohabitating households. These results suggest there is a possible bias in self-reported food security status based on gender, where females tend to report more severe issues of food insecurity than males.

By design, if a household receives monthly SNAP benefits to assist with food supplies, it should indicate relief of food insecurity. Our analyses support this idea; receipt of SNAP partially reduced food insecurity for lesbian and bisexual females. However, this finding may be more nuanced. In their study assessing the benefits of SNAP on food security, Gregory and Smith [14] determined that responses to surveys that utilize a 30-day food security assessment (including NHIS) are influenced by the date when SNAP benefits are received by beneficiaries. The probability of being classified as food insecure increased by 11% near the end of or at the very beginning of a benefit month [14]. Therefore, it is possible that respondents who were classified as food insecure in our study could have been unjustly placed in that category based solely on when their last SNAP benefit allocation was received. Meaning, SNAP may relieve food insecurity, but because food security was unintentionally assessed at the end of the month, when SNAP benefits may have run out, SNAP appeared to have less impact on food insecurity. Additionally, other studies that utilized a 12-month food security assessment showed no differences in SNAP receipt and use by sexual orientation [8, 27].

Disparities in food insecurity by sexual orientation are concerning because food insecurity is a leading predictor of chronic disease [3, 28] and may be contributing to the documented disparities in chronic conditions among LGB people [29], including cardiovascular disease [30] and certain types of cancer [31]. Food insecurity may contribute to chronic disease through multiple channels including stress caused by the need to secure food. This idea is supported by preliminary evidence. Using population-based data, Henderson and colleagues [32] investigated if stress associated with not having enough money to buy food varied by sexual orientation and found that LGB adults were 49% more likely to report stress associated with securing food than heterosexual adults (OR 1.49; 95% CI = 1.08–2.07, *p* < .05). Such stress may be compounded for LGB adults given the documented levels of minority stress experienced by this population [33] and the impact of this stress on mental health disparities experienced by sexual minorities [34]. This may be particularly true for bisexual adults, who reported the highest levels of food insecurity in this sample, given the stigmatization associated with bi-erasure and biphobia [35].

### Limitations

Limitations of the current study relate to the data elements collected. Given the small sample of LGB adults (*n* = 201 lesbian, *n* = 253 gay, *n* = 320 bisexual), we were unable to disaggregate and analyze the data by LGB status. All persons who identified as lesbian, gay, or bisexual were listed under a combined “LGB” variable (*n* = 774) for data analysis. Additionally, there were no questions concerning transgender-inclusive gender identity. Indeed, the underrepresentation of sexual and gender minorities is prominent in U.S. government data collection and oversampling within these populations can be beneficial for future data collection [36, 37]. Although NHIS survey sample weights were used, the data are from 2017 and may not be as representative of the population as the 2020 NHIS survey data. Additionally, omitted variable bias could limit the internal validity of results. Only individuals who applied for and received SNAP were classified as having receiving SNAP benefits. It is possible our findings are limited by the fact that not all respondents in need of SNAP applied for or received SNAP benefits. The self-report nature of the food insecurity and SNAP beneficiary variables are also a limitation. NHIS by design utilizes a 30-day food security assessment. While the 30-day assessment is an approved measurement duration by USDA standards [5], utilizing a 12-month food security assessment can provide a more accurate portrayal of prolonged food insecurity among survey respondents [38]. The utilization of a 30-day food security assessment tool limits our ability to make conclusions about long-term food security status among NHIS respondents who receive SNAP, as well as the timing of SNAP benefits in relation to experiences of food insecurity. Finally, our analyses were not capable of addressing intersectional marginalized identities that may elevate risk for food insecurity among LGB people who hold multiple marginalized identities [27, 39].

### Strengths

Despite the limitations described, our findings add to the growing empirical evidence that documents food insecurities among sexual minority adults, an understudied population. The population-based methods are rigorous and adhere to the best practices for investigating patterns in health using health surveillance data sources. In addition, we were able to describe food security among sexual minority adults using the gold standard, multi-item USDA module. Finally, our results further reiterate a need for sexual orientation to be included in nationally representative, 12-month federal food security measures so that public health professionals can have a more robust representation of long-term food insecurity issues among sexual minority populations.

## Conclusion

The COVID-19 pandemic exacerbatted the number of food insecure people in the U.S. [40]. As food insecurity continues to affect more households in the U.S., understanding the differential impact of food insecurity and programs designed to lessen its toll (e.g., SNAP) on minoritized populations, such as sexual minorities, is key to alleviating these potential disparities.

Our results indicate a partial mediation of the association between sexual orientation and food insecurity for lesbian and bisexual females when SNAP was received. This result indicates that assistance from federal food programs has the capability to lessen rates of food insecurity in marginalized populations. During the COVID-19 pandemic, food insecurity rates were expected to reach never-before-seen proportions. However, the federal response to swiftly increase SNAP allocations during the pandemic (P-EBT) to in-need families mitigated a potential food insecurity crisis [41]. The increase in SNAP allocations to quell the anticipated sharp rise in food insecurity during the COVID-19 pandemic suggests that sustained increases to monthly SNAP allocations may help to alleviate long-term food insecurity among lower-income populations. Our findings provide critical information to guide and support the development of services and interventions to address food insecurity experienced by sexual minorities, as well as the development of policies to aid this population.

## Data Availability

The datasets analyzed during the current study are available in the Centers for Disease Control and Prevention, National Center for Health Statistics, National Health Interview Survey repository, https://www.cdc.gov/nchs/nhis/data-questionnaires-documentation.htm.

## Abbreviations

FPL: Federal Poverty Level
LGB: Lesbian, Gay, and Bisexual
NHIS: National Health Interview Survey
NHANES: National Health and Nutrition Examination Survey
SNAP: Supplemental Nutrition Assistance Program
USDA: United States Department of Agriculture
